# Carnosol Is a Novel Inhibitor of p300 Acetyltransferase in Breast Cancer

**DOI:** 10.3389/fonc.2021.664403

**Published:** 2021-05-13

**Authors:** Halima Alsamri, Hussain El Hasasna, Bincy Baby, Aysha Alneyadi, Yusra Al Dhaheri, Mohammed Akli Ayoub, Ali H. Eid, Ranjit Vijayan, Rabah Iratni

**Affiliations:** ^1^ Department of Biology, College of Science, United Arab Emirates University, Al Ain, United Arab Emirates; ^2^ Department of Basic Medical Sciences, College of Medicine, QU Health, Qatar University, Doha, Qatar

**Keywords:** p300, acetyltransferase activity, reactive oxygen species, proteasome degradation, breast cancer, docking

## Abstract

Carnosol, a natural polyphenol abundant in edible plants such as sage, rosemary, and oregano, has shown promising anticancer activity against various types of cancers. Nonetheless, very little is known about its molecular mechanism of action or its downstream target(s). We have previously shown that carnosol inhibits cellular proliferation, migration, invasion, and metastasis as well as triggers autophagy and apoptosis in the highly invasive MDA-MB-231 breast cancer cells. Here, we report that carnosol induces histone hypoacetylation in MDA-MB-231 and Hs578T breast cancer cells. We show that, while carnosol does not affect HDACs, it promotes a ROS-dependent proteasome degradation of p300 and PCAF histone acetyl transferases (HATs) without affecting other HATs such as GCN5 and hMOF. Carnosol-induced histone hypoacetylation remains persistent even when p300 and PCAF protein levels were rescued from degradation by (i) the inhibition of the proteasome activity by the proteasome inhibitors MG-132 and bortezomib, and (ii) the inhibition of ROS accumulation by the ROS scavenger, N-acetylcysteine. In addition, we report that, in a cell-free system, carnosol efficiently inhibits histone acetyltransferase activity of recombinant p300 but not that of PCAF or GCN5. Molecular docking studies reveal that carnosol inhibits p300 HAT activity by blocking the entry of the acetyl-CoA binding pocket of the catalytic domain. The superimposition of the docked conformation of the p300 HAT domain in complex with carnosol shows a similar orientation as the p300 structure with acetyl-CoA. Carnosol occupies the region where the pantetheine arm of the acetyl-CoA is bound. This study further confirms carnosol as a promising anti-breast cancer therapeutic compound and identifies it as a novel natural p300 inhibitor that could be added to the existing panel of inhibitors.

## Introduction

Reversible acetylation of histone and non-histone proteins is one of the major post-translational mechanisms involved in regulating gene expression as well as protein activity (1, 2). Acetylation on lysine residues was identified in many proteins involved in diverse cellular processes, such as differentiation, proliferation, signal transduction, cell cycle, apoptosis, DNA repair, autophagy, senescence, and cytoskeleton dynamics (3, 4). Furthermore, histone lysine acetylation is one of the main epigenetic modifications that play a role in chromatin remodeling and dynamics with an impact on gene expression and transcriptional activities (5). To maintain cellular homeostasis, protein acetylation is tightly regulated in a cell by the balanced action of two classes of enzymes, histone acetyltransferases (HATs) and histone deacetylases (HDACs) (6, 7). HATs regulate fundamental cellular processes in the cell and are implicated in the etiology of several diseases. Deregulated HAT activity is particularly linked to cancer formation and progression (1, 8), which is observed in several solid tumors, including prostate (9, 10), colon (11, 12), and breast (13, 14) cancers. Thus, HATs and HDACs are potential drug targets, and the restoration or modification of their activity could be beneficial for both the management and treatment of cancer (1, 8).

In mammalian cells, the HAT family comprises three subfamilies: the GNAT, the MYST, and the p300/CBP proteins (1). p300 is a transcriptional coactivator, favoring transcription, and a lysine acetyl-transferase is involved in regulating a wide range of biological processes (9). An increasing body of evidence suggests that p300 may be an important player in cancer (15). However, its precise role is not clear and may depend on the physiologic background of the tumor (9). Interestingly, evidence indicates that p300 can function both as a tumor suppressor and as an oncoprotein in cancer (15). In fact, in invasive breast cancer, the high expression of p300 was found to correlate with recurrence and poor patients’ prognosis (16, 17). Moreover, p300 was reported to be involved in breast cancer progression by promoting cellular invasion and cellular survival (18). Thus, targeting p300 in invasive breast cancer may be a promising approach for the development of novel anticancer therapy. Although HDAC inhibitors are being extensively explored as cancer therapeutics compounds and have been progressed through different stages of preclinical and clinical development (19), to date, only a few potent and specific acetyltransferase inhibitors have been identified (2).

PCAF (p300/CBP-associated factor), another histone acetyltransferase and a component of several protein complexes was shown to play distinct roles in cancer, such as tumor suppressors, oncogenes, and transcription factors that control cell differentiation and cell cycle (20). For instance, PCAF can acetylate NF-κB in response to inflammation and immunity (21), or it can acetylate p53 in response to DNA damage leading to cell cycle arrest (22). Also, PCAF has been reported to function as a tumor repressor by promoting apoptosis in hepatocellular carcinoma (23) and inhibit the cell cycle in gastric cancer cells (24). On the other hand, PCAF was found to promote tumorigenicity in lung adenocarcinoma *via* acetylating EZH2 (25) and enhance cellular proliferation of glioblastoma *via* Akt1 acetylation (26).

Recent experimental evidence supports the idea that phytochemicals directly influence epigenetic mechanisms in humans (27, 28). It may lead to increased sensitivity of cancer cells to conventional therapy and thus inhibition of tumor growth. Various phytochemicals have been identified as modulators of the acetylation state of histones or affect the activities of HATs and/or HDACs (29). Curcumin (30), anacardic acid (31), garcinol (32), epigallocatchechin 3-gallate (33), and plumbagin (34) have been shown to possess specific HAT inhibitor activity. Among these, curcumin was found to be the only known p300-specific natural inhibitor, both *in vitro* and *in vivo*, which is also cell permeable (30, 35). Interestingly, curcumin’s specific binding on p300/CBP led to a conformational change resulting in a decrease in the binding efficiency of histones H3, H4, and acetyl CoA (35).

Carnosol is a major compound in many culinary plants such as sage, rosemary, and oregano with anti-inflammatory, anti-oxidant, anti-microbial, and anticancer properties (36). In recent years, carnosol has shown promising anticancer activities *in vitro* and *in vivo* against several human cancer, including colon (37, 38), breast (39), gastric (40), and prostate (41) cancer.

Here we report that carnosol induced histone hypoacetylation in the highly invasive triple negative breast cancer (MDA-MB-231) cells. We found that carnosol specifically targeted p300 and PCAF acetyltransferase to proteasome degradation through a ROS-dependent mechanism. Also, we show that carnosol specifically inhibits p300 acetyl transferase activity by competing with acetyl-CoA for the HAT catalytic domain.

## Materials and Methods

### Cell Culture, Chemicals, and Antibodies

Human breast cancer cells MDA-MB-231(Cat. # 300275) were purchased from Cell Line Service (CLS)-GmbH and Hs578T (cat# HTB-126) were purchased from ATCC-USA. Both cell lines were maintained in Dulbecco’s modified eagle medium (DMEM) (Cat. # 03640, Gibco, Life Technologies, Rockville, UK). T47D was maintained in RPMI (Cat. # 00506 Gibco, Life Technologies, Rockville, UK). All media were complemented with 10% fetal bovine serum (FBS) (Cat. # 02187 Gibco, Life Technologies, Rockville, UK) and 100 U/ml penicillin streptomycin glutamine (Cat. # 01574 Gibco, Life Technologies, Rockville, UK). Carnosol (Cat. # C9617), N-Acetyl-L-cysteine (NAC) (Cat. # A9165), caspase inhibitor (Cat. # 627610), 3-MA an autophagy inhibitor (Cat. # 189490), anti-histone H4 antibody (Cat. # 07-108), anti-acetyl-Histone H4 antibody (Cat. # 382160), anti-acetyl-histone H3 antibody (Cat. # 06-599), and core histone proteins (Cat. # 13-107) were purchased from Sigma Aldrich. Anti-KAT/MYST1/MOF antibody (Cat. # ab72056), anti-histone H3 antibody (Cat. # ab201456), anti-histone H4 (acetyl K16) antibody (Cat. # ab109463), anti-histone H3 (acetyl K56) (Cat. # ab76307) antibody, recombinant human STAT3 protein (Cat. # ab43618), recombinant human histone H3 protein (Cat. # ab198757), and chloroquine diphosphate (Cat. # ab142116) were purchased from Abcam. p300 (F-4) antibody (Cat. #sc-48343), GAPDH antibody (Cat. # sc-25778), GCN5 antibody (Cat. # sc-20698), PCAF antibody (Cat. # sc-13124), HDAC1 antibody (Cat. # sc-7872), HDAC2 antibody (Cat. # sc-9959), *β*-actin antibody (Cat. # sc-47778), goat anti-mouse IgG-HRP antibody (Cat. # sc-2005), goat anti-rabbit IgG-HRP antibody (Cat. # sc-2004), and acetyl coenzyme A sodium salt (Cat. # sc-210745A) were purchased from Santa Cruz Biotechnology. Acetyl-histone H3 (Lys9) antibody (Cat. # 9649), acetylated-lysine antibody (Cat. # 9441), acetyl-histone H4 antibody (Cat. # 382160), acetyl-histone H3 (Lys14) antibody (Cat. # 7627), acetyl-CBP (Lys1535)/p300 antibody (Cat. # 4771), MG-132 (Cat. # 2194), Bortezomib (Cat. # 2204S), TSA (Cat. # 9950S), Bortezomib (Cat. # 2204) MG-132 (Cat. # 2194), and cycloheximide (Cat. # 2112) were purchased from Cell Signaling. Human recombinant p300 (Cat. # BML-SE451-0100), human recombinant CREB binding protein (Cat. # BML-SE452-0100), human recombinant GCN5 (Cat. # BML-SE272-0050), and human PCAF/histone H3 (human) (Cat. # BML-P271-0500) were purchased from Enzo.

### RNA Extraction, RT-PCR, and qRT-PCR

Total RNA from vehicle- or carnosol-treated MDA-MB-231 cells were prepared using Trizol reagent as described by the manufacturer (Life Technologies, Inc., Rockville, UK). For RT-PCR, RNA expression of p300 and PCAF was determined using the Qiagen OneStep RT-PCR kit (Qiagen, Germany) according to the manufacturer’s instruction. Equal amounts of RNA (500 ng) were used as templates in each reaction. The PCR products were separated by 1.5% agarose gel and visualized by ethidium bromide staining. Representative results from two independent experiments are shown. The qRT-PCR amplification was performed using Bio-Rad CFX96 Real-Time PCR System C1000 Thermal Cycler (Bio-Rad, Hercules, California, USA) triplicates. The reaction mixture (20 μl) contains 100 ng total RNA, 10  μl 2× GoTaq qPCR Master Mix, 0.4 μl 50× GoScript RT Mix for one-Step qRT-PCR (Promega, Madison, Wisconsin, USA), 0.3 μM each specific sense and antisense primer. The reaction condition was as follows: 40°C for 15 min for the reverse transcription (RT) followed by 95°C for 10 min of RT inactivation, 40 cycles of 95°C for 10 s, 58°C for 30 s, and 72°C for 30 s. Expression levels were calculated by the comparative cycle threshold method, and normalization to the control was performed. RT-PCR and qRT-PCR were performed using GAPDH as an endogenous reference for normalization. The sequences of specific primers were as follows: p300 sense, 5′-TTACAGCGACAAGAAGTGG-3′, and antisense, 5′-CCCTCAGTGAAGCGGTACAT-3′; p300 sense 5′-CAATGAGATCCAAGGGGAGA-3′, and antisense 5′-ATGCATCTTTCTTCCGCACT-3′; PCAF sense, 5′-CTGGAGGCACCATCTCAACGAA-3′ and antisense, 5′-ACAGTGAAGACCGAGCGAAGCA-3′; GAPDH sense, 5′-GGCCTCCAAGGAGTAAGACC-3′, and antisense: 5′-AGGGGTCTACATGGCAACTG-3′.

### Whole Cell Extract and Western Blotting Analysis

Cells (1.8 × 10^6^) were seeded per 100 mm culture dish and cultured 24 h before treatment. After incubation, cells were washed twice with ice-cold PBS, scraped, pelleted, and lysed in RIPA buffer (Pierce) supplemented with protease inhibitor cocktail (Roche) and phosphatase inhibitor (Roche). After incubation for 30 min on ice, cell lysates were centrifuged at 14,000 rpm for 20 min at 4°C. The protein concentration of lysates was determined by the BCA protein assay kit (Thermo Scientific), and the lysates were adjusted with lysis buffer. Aliquots of 25 µg of total cell lysate were resolved onto 6–15% SDS-PAGE. Proteins were transferred to nitrocellulose membranes (Thermo Scientific) and blocked for 1 h at room temperature with 5% non-fat dry milk in TBST (TBS and 0.05% Tween 20). Incubation with specific primary antibodies was performed in blocking buffer overnight at 4°C. Horseradish peroxidase-conjugated anti-IgG was used as a secondary antibody. Immunoreactive bands were detected by ECL chemiluminescent substrate (Thermo-Scientific), and chemiluminescence was detected using the LiCOR C-DiGit blot scanner. Western Blots were repeated three times using different protein preparations each time. Quantification was carried out using ImageJ software.

### Histone Acetyltransferase Activity Using a Colorimetric Assay

HAT activity was measured using the commercially available colorimetric HAT activity assay kit (Abcam). Briefly, 50 µg of HeLa cell nuclear extract (Santa Cruz Biotechnology) was incubated, with or without carnosol, in HAT assay buffer according to the manufacturer’s instructions. Absorbance was measured at 450 nm. Experiments were carried out in triplicate and repeated three times. Data are represented as mean values ± SEM.

### *In Vitro* Histone Acetyl Transferase Activity Assay

One hundred nanograms of recombinant HATs (p300, PCAF or GCN5) was incubated in the presence of a HAT assay buffer (50mM tris pH8.0, Glycerol 10%, 0.1 mM EDTA, 1 mM dithiotheithol, 1 mM PMSF), 400 nM trichostatin A, 20 µM Acetyl-CoA in the presence of DMSO, as control, or carnosol for 1 h at 30°C. The reaction was stopped by the addition of SDS-loading buffer. HAT activity was determined by Western blotting using an antibody specific for acetylated histones. Experiments were carried out in triplicate and repeated three times. Data are represented as mean values ± SEM.

### Molecular Docking

Three-dimensional (3D) X-ray crystallographic structures of histone acetyltransferases p300 were retrieved from the Protein Data Bank (PDB) (42) (**Table 1**). These structures were pre-processed using the protein preparation wizard of Schrödinger Maestro using the default settings to prepare them for molecular docking (43–45). Subsequently, energy minimization was done to obtain geometrically stable structures (43). The two-dimensional (2D) structure of carnosol and acetyl CoA were obtained from PubChem (46). For a comparative analysis, 2D structures of known histone acetyltransferases inhibitors—CPTH2 and C646—were also retrieved. Schrödinger LigPrep was used to convert these to 3D and prepare them for docking using the default protocol (43, 47). Acetyl CoA binding region of each of the targets was identified, and a receptor grid was generated around this region by incorporating all residues in the binding site. No constraints were used in the generation of the receptor grids. The OPLS 2005 force field was applied to model the protein, ligand, and interactions (48). Grid-based docking was carried out to predict the binding pose and docking score of the compounds using Schrödinger Glide in extra precision (XP) mode (49). The ligands were flexibly docked into the active site of the histone acetyltransferase structures. Post docking, protein–ligand interactions, such as hydrogen bond, hydrophobic interactions, *π*–*π* stacking and cation–*π* interactions and XP GlideScore docking score were analyzed. The binding energy of the docked poses were calculated using Schrödinger Prime based on the molecular mechanics generalized Born surface area (MM-GBSA) approach (44, 45).

**Table 1:** p300 structures used for molecular docking.

Protein	PDB ID	Resolution (Å)
p300	4PZR	2.1
	4PZS	1.94
	4PZT	2.8

### Statistical Analysis

Statistical analyses were performed using SPSS version 21 for PC (IBM Corp.). Data were reported as group mean ± SEM. The data were analyzed *via* one-way ANOVA. Significance for all statistical comparisons was set at p < 0.05.

## Results

### Carnosol Induced Histone Hypoacetylation in Breast Cancer Cells

**Figure 1:**
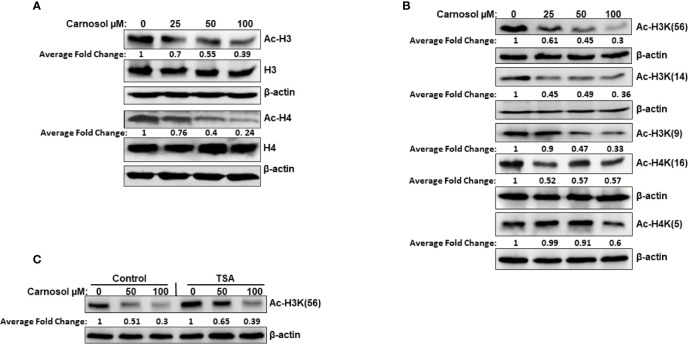
Carnosol induces histone hypoacetylation in vivo in MDA-MB-231 breast cancer cells. **(A)** Carnosol induces overall histone H3 and H4 hypoacetylation. MDA-MB 231 cells were treated with vehicle (DMSO) or increasing concentrations of carnosol then whole-cell extracts were subjected to Western blot analysis for the acetylated histone H3 and histone H4. **(B)** Carnosol induces histone hypoacetylation at specific residues. whole-cell extracts were subjected to Western blot analysis for the acetylated H3K56, H3K14, H3K9, H4K16, and H4K5. **(C)** HATs, and not HDAC, are involved in carnosol-induced histone hypoacetylation. Trichostatin A (TSA) has no effect on carnosol-induced histone H3K(56) hypoacetylation, one of p300 substrates. Cells were pre-treated with or without TSA (400 nM) for 4 h and then treated with carnosol (50 and 100 µM). Whole cell lysate was resolved on 15% SDS gel and probed with acetyl-H3K56 antibody. Data shown are representative of three independent experiments carried at different times using different protein preparations. The intensities of the bands were quantified using the ImageJ software (National Institute of Health, USA).

We have examined the overall acetylation status of histone H3 and histone H4 in carnosol-treated breast cancer cells. Our data showed that carnosol induced an overall decreased in the acetylation level of histone H3 and H4 in MDA-MB-231 (**Figure 1A**) and Hs578T (**Supplementary Figure 1**) triple negative breast cancer cells. Next, we examined the acetylation status of specific lysine residues known to be substrates for acetylation by HATs by using antibodies directed explicitly against acetylated H3K56, H3K14, H3K9, H4K5, and H4K16. Carnosol showed a differential effect on the acetylation of different residues (**Figure 1B**). While carnosol had little impact on acetylation of H4K5 and H4K16, it dramatically reduced the level of acetylation of H3K56, H3K9, and H3K14 in MDA-MB-231 cells (**Figure 1B** and **Supplementary Figure 1**). To rule out that the carnosol-induced histone hypoacetylation is a result of upregulated histone deacetylase (HDAC) activity, MDA-MB-231 cells were first pre-treated with the HDAC inhibitor, TSA, and then treated with carnosol for 24 h. As shown in **Figure 1C**, inhibition of HDAC activities by TSA had no effect on carnosol-induced histone hypoacetylation of histone H3K56. Hence, our results suggest that carnosol might induce histone hypoacetylation in breast cancer by affecting the activity of histone acetyl transferase(s) in breast cancer cells. Also, considering that carnosol induced histone hypoacetylation at specific residues suggests that it exerts its effect on particular HATs.

### Downregulation of p300 and PCAF Protein Levels in Carnosol Treated Breast Cancer Cells

**Figure 2:**
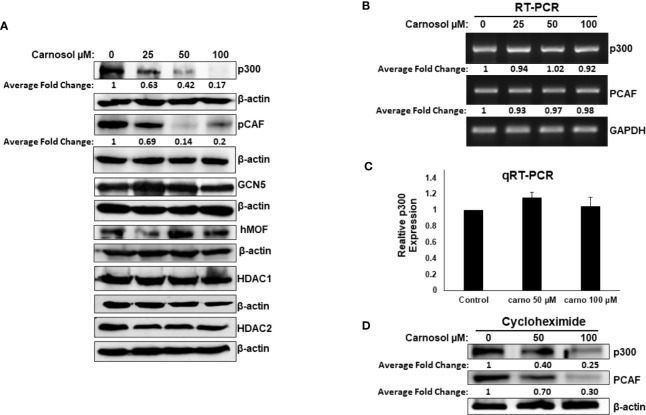
Carnosol downregulates p300 and PCAF in MDA-MB-231 cells. **(A)** Downregulation of p300 and PCAF protein levels in carnosol-treated MDA-MB-231 cells. MDA-MB-231 cells were treated with vehicle (DMSO) or indicated concentrations of carnosol and whole cell lysates were resolved in 6% SDS-PAGE and subjected to Western blot analysis for the indicated proteins. Data shown are representative of three independent experiments. **(B, C)** carnosol does not affect the levels of p300 and PCAF mRNA transcripts. Total RNA from carnosol-treated and untreated cells were used to amplify the PCAF and p300 transcripts by RT- and qRT-PCR. RT- and qRT-PCR were repeated twice. **(D)** Downregulation of p300 and PCAF proteins by carnosol in cycloheximide-pre-treated cells. Cells were pre-treated with cycloheximide, an inhibitor of *de novo* protein synthesis, for 2 h and then treated with or without carnosol (50 and 100 µM). Whole cell lysates were collected and subjected to Western blot for p300 and PCAF. The intensities of the bands of Wester blots were quantified using the ImageJ software (National Institute of Health, USA).

We next examined Western blotting, the expression level of proteins p300, PCAF, GCN5, and hMOF histone acetyl transferases. We found that carnosol induced a dramatic decrease in a concentration-dependent manner of p300 and PCAF protein level in MDA-MB-231 (**Figure 2A**) and Hs578T (**Supplementary Figure 2**) cells. The level of GCN5 and hMOF, on the other hand, remained unchanged in carnosol-treated cells. As expected, carnosol did not affect the expression level of histone deacetylases (HDAc1 and HDAC), as shown in **Figure 2A**. Our results indicate that carnosol specifically downregulates p300 and PCAF in breast cancer cells. To elucidate the mechanism by which carnosol downregulated p300 and PCAF, we first examined level of p300 and PCAF mRNA transcript in control and carnosol-treated MDA-MB-231 cells. RT-PCR revealed no significant changes in mRNA levels of both transcripts between control and carnosol-treated cells (**Figure 2B**). This result was further confirmed by qRT-PCR for both p300 (**Figure 2C**) and PCAF (data not shown). We next sought to test whether carnosol influences p300 and PCAF stability. For this, cells were first pre-treated with cycloheximide, an inhibitor of eukaryotic translation, and then treated with carnosol. As shown in **Figure 2D**, carnosol-induced p300 and PCAF decrease occurred in the presence of cycloheximide. Altogether, our data strongly suggest that carnosol downregulated p300 and PCAF protein level at the post-translational level, possibly by targeting them for degradation. In addition, we hypothesized that histone hypoacetylation in response to carnosol is a result, at least partly, of p300 and PCAF downregulation.

### Carnosol Induces ROS-Dependent Proteasome Degradation of p300 and PCAF

**Figure 3:**
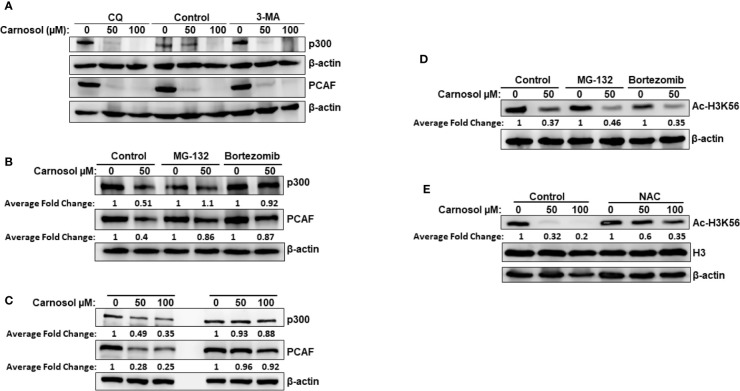
Carnosol promotes a ROS-dependent proteasome degradation of p300 and PCAF in MDA-MB-231 cells. **(A)** Western blot analysis of p300 and PCAF protein level in MDA-MB-231 cells pre-treated with the autophagy inhibitors 3-MA and CQ. Cells were pretreated with or without 3-MA (50 mM) and CQ (50 μM) for 1 h and before addition of carnosol and incubation for another 24 h. **(B)** Carnosol induces proteasome-dependent degradation of p300 and PCAF. MDA-MB-231 were pre-treated for 1 h with or without the proteasome inhibitors MG-132 (15 μM) or Bortezomib (25 nM) before treatment with carnosol. **(C)** ROS-dependent proteasome degradation of p300 and PCAF. MDA-MB-231 were pre-treated for 1 h with the ROS scavenger, NAC, before adding carnosol (50 and 100 μM). Whole cells lysates were resolved on 6% SDS-PAGE and analyzed by Western blot for p300 and PCAF proteins. **(D, E)** Rescue of p300 and PCAF, by proteasome inhibitors **(D)** and ROS scavenger **(E)** did not restore the acetylation of H3K56, a preferred p300 substrate. In all Western blot experiment, 30 μg of whole cell extracts was loaded in each lane. The intensities of the bands were quantified using the ImageJ software (National Institute of Health, USA). Data shown are representative of three independent experiments.

We have previously reported that carnosol triggered autophagy in MDA-MB-231 breast cancer cells (39). We, therefore, decided to examine whether p300 and PCAF were degraded by autophagy. We found that blockade of early stage autophagy (autophagosome formation) by 3-MA and late stage autophagy (autophagolysosome formation) by CQ failed to rescue p300 and PCAF protein levels (**Figure 3A**) and, therefore, excluding autophagolysosomal degradation as the mechanism responsible for the decrease of the two HATs. We next tested whether proteasomal activity is involved in the carnosol-mediated decrease of the two proteins. MDA-MB-231 cells were first pre-treated for 1 h with the proteasome inhibitor MG-132 or bortezomib before carnosol was added. As shown in **Figure 3B**, both proteasome inhibitors efficiently restored the levels of p300 and PCAF proteins to levels comparable to untreated cells. This result clearly indicated that carnosol targets p300 and PCAF to proteasome degradation. We recently reported that carnosol promoted proteasome degradation of STAT3 protein through a ROS-dependent mechanism (38). This prompted us to test whether ROS production accounts for the degradation of p300 and PCAF. Blockade of ROS accumulation by the ROS scavenger N-acetylcysteine (NAC) efficiently blocked carnosol-mediated proteasome degradation of the two HATs in both MDA-MB-231 (**Figure 3C**) and Hs578T (**Supplementary Figure 3A**) cells. Altogether, our data demonstrate that carnosol targets p300 and PCAF to a ROS-dependent proteasome degradation.

Having demonstrated that proteasome inhibition (**Figure 3B**) and blockade of ROS accumulation (**Figure 3C**) efficiently restored the levels of p300 and PCAF to those observed in the untreated control cells, we next sought to test whether this was also associated with restoration the histone acetylation status. Strikingly, we found that inhibition of HAT degradation by proteasome inhibitors (**Figure 3D**) or ROS scavenger (**Figure 3E**) failed to restore the histone acetylation of H3K56, a preferred p300 substrate, in MDA-MB-231. Also, NAC had no effect on carnosol-induced hypoacetylation of H3K56 in Hs578T cells (**Supplementary Figure 3B**). Our data strongly suggested that, in addition to promoting p300 and PCAF degradation, carnosol might also exert its anti-breast cancer effect through direct inhibition of HAT activity.

### Carnosol Inhibits p300 Acetyltransferase Activity *In Vitro*

**Figure 4:**
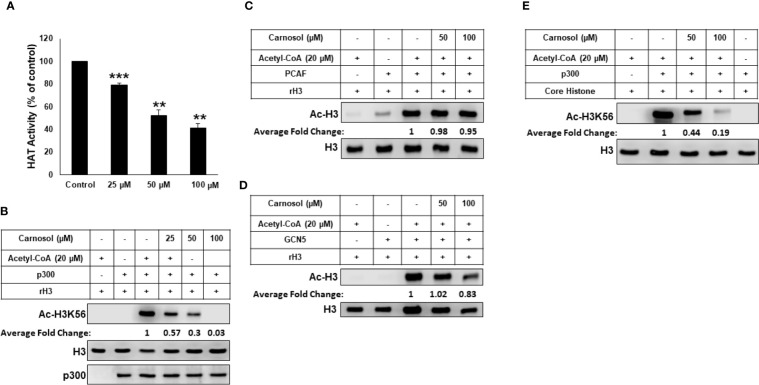
Carnosol is a specific p300 inhibitor in vitro. **(A)** Carnosol inhibit histone acetylation *in vitro*. HAT assay with HeLa nuclear extract in the presence of increasing concentrations of carnosol. Values represented in percent were calculated from three independent experiments carried out in triplicate and are represented as mean ± SEM. (**p < 0.005, ***p < 0.001). **(B–D)** Carnosol inhibits P300 **(B)** but not PCAF **(C)** or GCN5 **(D)**. HAT assay was performed with p300 catalytic domain (aa 1,284**–**1,673), recombinant PCAF and recombinant GCN5, recombinant H3 with or without increasing carnosol. HAT activity was measured by Western Blotting scoring for acetylated histone H3. **(E)** HAT assay was performed with recombinant p300 catalytic domain (aa 1,284**–**1,673) and core histone with or without carnosol and HAT activity was measured as in **(B)**. Data shown for all HAT assays are representative of three independent experiments. The intensities of the bands were quantified using the ImageJ software (National Institute of Health, USA).

We tested the ability of carnosol to inhibit lysine acetyltransferase activity in a cell-free system using HeLa nuclear extract (NE) as source for HATs or recombinant p300. We found that carnosol significantly inhibited HAT activity in a concentration-dependent manner in both NE- (**Figure 4A**) and recombinant p300-based (**Supplementary Figure 4**) HAT assay, suggesting that carnosol is an inhibitor of acetyltransferase activity. We next sought to determine whether carnosol is a specific or pan-HAT inhibitor. Toward this, we used *in vitro* acetylation assay with purified histone H3 and recombinant p300, PCAF and GCN5 acetyltransferases. We found that carnosol efficiently inhibited p300-dependent histone acetylation (**Figure 4B**) while it showed no significant detectable inhibition of PCAF (**Figure 4C**) and GCN5-dependent histone acetylation (**Figure 4D**). We have also tested the ability of carnosol to inhibit p300 HAT activity using core histones as a substrate. As shown in **Figure 4E**, carnosol also effectively inhibited the acetylation of H3K56 in this assay. Altogether our results identified carnosol as a new natural specific inhibitor of p300 activity.

### Carnosol Inhibits the Acetyltransferase Activity of p300 by Obstructing the Entrance of Acetyl-CoA Binding Pocket of the Catalytic Domain

**Figure 5:**
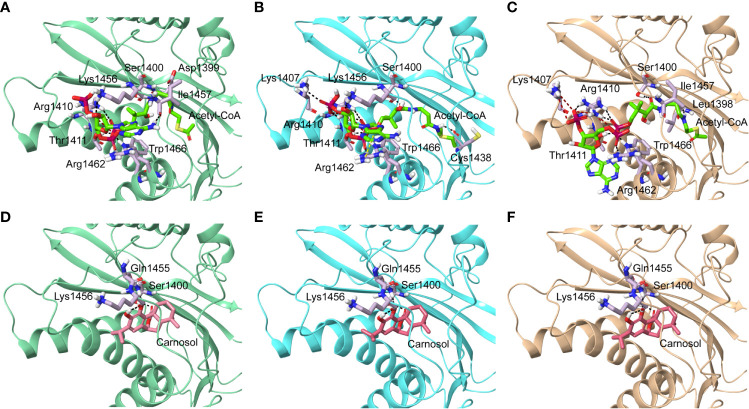
Molecular docking of acetyl-CoA and carnosol in the active site of p300. Polar interactions of acetyl-CoA docked in the active site of the p300 in the structure with Protein Data Bank ID: **(A)** 4PZR, **(B)** 4PZS, and **(C)** 4PZT. Polar interactions of carnosol docked in the active site of the p300 in the structure with Protein Data Bank ID: **(D)** 4PZR, **(E)** 4PZS, and **(F)** 4PZT. The protein structure is shown in cartoon representation (4PZR in green, 4PZS in cyan, and 4PZT in brown), acetyl CoA is shown in green stick representation, and carnosol is shown in pink stick representation. Protein residues that interact with the docked ligand are shown in lilac stick representation. Hydrogen bonds are represented by black dashed lines.

We next carried out *in silico* molecular docking studies to test the potential affinity of carnosol toward p300 lysine acetyltransferase. We first carried out a docking analysis of the acetyl-CoA-p300 complex. The acetyl-CoA binding site of p300 has a deep hydrophobic cavity that extends into the interior of the protein. The adenosine ring of the acetyl-CoA was sandwiched between Lys1456 and Arg1462, and the pantetheine arm embedded itself deep inside the hydrophobic tunnel and made extensive interactions with the substrate binding loop. The deep hydrophobic cavity is lined by hydrophobic residues Ile1395, Tyr1397, Leu1398, and Tyr1414 that interacted with the pantetheine arm. Arg1410 formed critical hydrogen bonds with the phosphate groups. In the case of acetyl-CoA in the 4PZR structure, Trp1436, Ala1437, Cys1438, Pro1439, Pro1440, Tyr1446, and Pro1458 present in the substrate-binding loop formed hydrophobic interactions with the pantetheine arm. The methyl moiety of the acetyl group was accommodated by the hydrophobic residues Ile1395, Tyr1397, Leu1398, and Cys1438 (**Figure 5A**). In the 4PZS structure with acetyl-CoA, the pantetheine arm bound deep inside the cavity by forming interactions with the hydrophobic residues in the substrate-binding loop such as Trp1436, Ala1437, Pro1439, Pro1440, Tyr1446, and Ile1457, and Pro1458 (**Figure 5B**). Similarly, in 4PZT structure with acetyl-CoA, the pantetheine arm made extensive hydrophobic interactions with the substrate-binding loop by interacting with Trp1436, Ala1437, Cys1438, Pro1439, Pro1440 and Tyr1446. Trp1436 and Tyr1467 (**Figure 5C**). Adenosine ring, ribose sugar, phosphates and the pantetheine arm formed hydrogen bonds with residues present in the N-terminal *β*-strands and also on the substrate-binding loop. Beside hydrophobic and hydrogen bond interactions, Arg1462 formed a cation–*π* interaction with the adenine ring of acetyl-CoA. The XP GlideScore, an empirical scoring function used by Glide to rank docked poses, produced scores ranging from −11.88 to −10.95 kcal/mol for the p300-acetyl-CoA complexes obtained from the three structures. The calculated MM-GBSA binding energy, an indicator of binding affinity ranged between −118.13 and −97.02 kcal/mol. Arg1410, Arg1462, and Lys1456 contributed the highest interaction energies to the binding affinity of acetyl-CoA with p300 (**Table 2**).

**Table 2:** Summary of binding energy and residues that interacted with ligands docked to p300 HAT.

PDB ID	Ligand	XP Gscore(kcal/mol)	Hydrogen bonds	Hydrophobic interactions	Pi-Pi	Pi-cation
4PZR	Acetyl coA	−11.17	Asp1399, Ser1400, Arg1410, Thr1411, Cys1438, Lys1456, Ile1457, Arg1462, Trp1466	Ile1395, Tyr1397, Leu1398,Tyr1414, Trp1436, Ala1437, Cys1438, Pro1439, Pro1440, Tyr1446, Pro1458, Leu1463, Phe1467		Arg1462
	carnosol	−9.19	Ser1400, Gln1455, Lys1456	Leu1398, Tyr1414, Cys1438, Pro1439, Pro1440, Tyr1446, Ile1457, Pro1458, Leu1463, Tyr1466		
	C646	−5.35	His1451, Gln1455, Trp1466	Leu1398, Tyr1414, Pro1440, Tyr1446, Pro1452, Ile1457, Pro1458, Leu1463		
	CPTH2	−6.14		Leu1398,Tyr1414, Ile1435, Trp1436, Ala1437, Cys1438, Pro1439, Pro1440, Tyr1446, Ile1457, Pro1458, Leu1463, Trp1466, Phe1467		
4PZS	Acetyl coA	−11.88	Ser1400, Lys1407, Arg1410, Thr1411,Cys1438, Lys1456, Arg1462, Trp1466	Tyr1394, Ile1395, Tyr1397, Leu1398, Tyr1414, Ile1435, Trp1436, Ala1437, Pro1439, Pro1440, Tyr1446, Ile1457, Pro1458, Leu1463, Phe1467		Arg1462
	carnosol	−9.04	Ser1400, Gln1455, Lys1456	Leu1398, Tyr1414, Cys1438, Pro1439, Pro1440, Tyr1446, Ile1457, Pro1458, Leu1463, Tyr1466		
	C646	−6.62	Arg1410, Lys1456, Arg1462	Leu1398, Tyr1414, Pro1439, Pro1440, Tyr1446, Ile1457, Pro1458, Leu1463, Tyr1466, Phe1467		Arg1410
	CPTH2	−6.86		Leu1398,Tyr1414, Ile1435, Trp1436, Ala1437, Cys1438, Pro1439, Pro1440, Tyr1446, Ile1457, Pro1458, Leu1463, Trp1466, Phe1467		
4PZT	Acetyl coA	−10.95	Leu1398, Ser1400, Lys1407, Arg1410, Thr1411, Ile1457, Arg1462, Trp1466	Ile1395, Tyr1397, Tyr1414, Ile1435, Trp1436, Ala1437, Cys1438, Pro1439, Pro1440, Tyr1446, Ile1457, Pro1458, Leu1463, Phe1467		Arg1462
	carnosol	−8.05	Ser1400, Gln1455, Lys1456	Leu1398, Tyr1414, Cys1438, Pro1439, Pro1440, Tyr1446, Ile1457, Pro1458, Leu1463, Tyr1466		
	C646	−8.74	Arg1410, Thr1411,Trp1436, Trp1466	Ile1395, Tyr1397, Leu1398, Ile1435, Ala1437, Cys1438, Pro1440, Tyr1446, Ile1457, Pro1458, Leu1463, Phe1467	Tyr1414, Trp1466	Tyr1394
	CPTH2	−6.72	Cys1438	Ile1395, Tyr1397, Leu1398,Tyr1414, Ile1435, Trp1436, Ala1437, Pro1439, Pro1440, Tyr1446, Pro1458, Leu1463, Trp1466, Phe1467	Trp1466	

We found that in the carnosol docked complexes, the substrate-binding loop of p300 encapsulated the molecule by forming hydrogen bonds and hydrophobic interactions. The active site is a deep hydrophobic tunnel lined by hydrophobic residues Leu1398, Tyr1414, Leu1463, and Trp1466. Carnosol occupied the region where the pantetheine arm of the acetyl-CoA binds and made extensive interactions with the substrate-binding loop. Residues Cys1438, Pro1439, Pro1440, Tyr1446, Ile1457, and Pro1458 interacted with the lactone moiety of carnosol. In all the three docked complexes, two hydroxyl groups of carnosol formed three hydrogen bonds with the backbone amino and carboxyl groups of Gln1455 and Lys1456 respectively and the lactone moiety formed a hydrogen bond with the hydroxyl group of Ser1400 (**Figures 5D–F**). p300 residues that interacted with carnosol are given in **Table 2**. Lys1456 contributed the largest interaction energy in the binding of carnosol with p300 structures. The GlideScore docking score ranged between −9.19 and −8.05 kcal/mol and the MM-GBSA binding energy ranged from −85.57 to −81.76 kcal/mol (**Table 2**).

**Figure 6:**
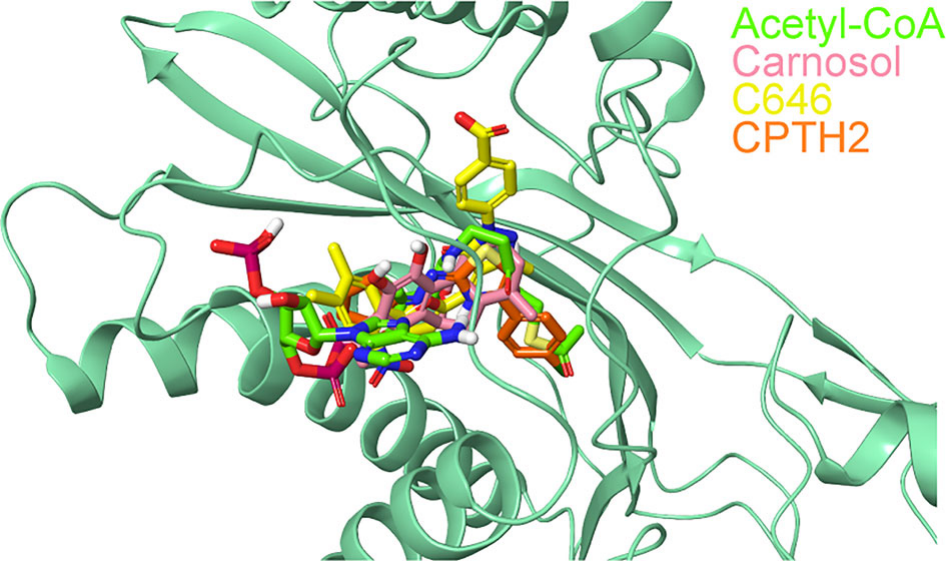
Superposition of docked ligands in the active site of p300. The protein structure is shown in green cartoon representation, and the docked ligands—acetyl-CoA (green), carnosol (pink), C646 (yellow) and CPTH2 (orange) —are depicted in stick representation.

The superimposition of the best docked conformation of carnosol in the p300 HAT domain showed similar orientation as the p300 structure with acetyl-CoA (**Figure 6**). Carnosol occupied the region where the pantetheine arm of the acetyl-CoA bound. Both, lactone moiety and pantetheine arm formed hydrogen bonds with Ser1400 and exhibited extensive interactions with the substrate-binding loop.

**Figure 7:**
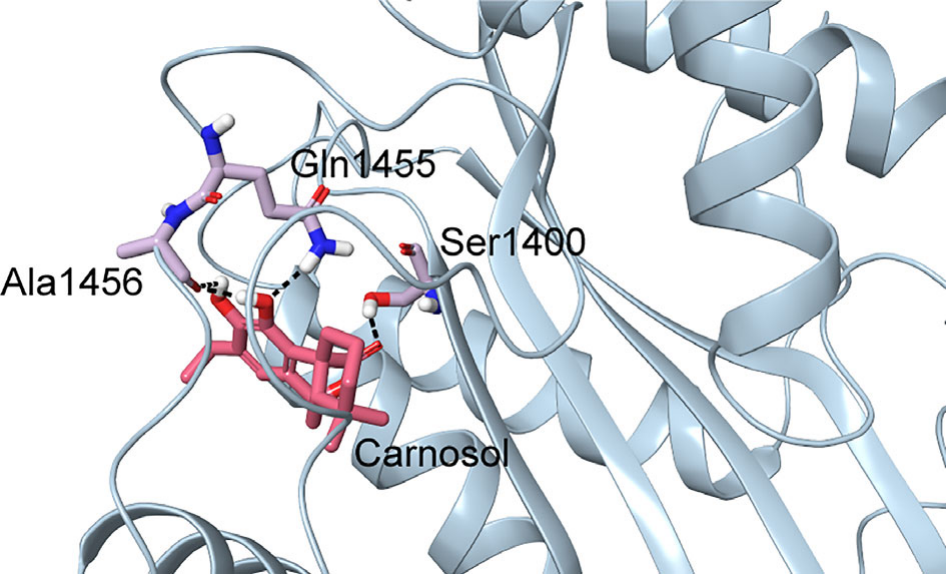
Docked pose of carnosol in the Lys1456Ala p300 structure. The protein structure is shown in blue cartoon representation, and carnosol is shown in pink stick representation. Protein residues that interact with carnosol are shown in lilac stick representation. Hydrogen bonds are represented by black dashed lines.

In p300, Lys1456 in the substrate-binding loop contributed the most interaction energy in the p300-carnosol complex. To characterize this interaction further, the lysine was mutated *in silico* to alanine (Lys1456Ala) and carnosol was docked. As evident from **Figures 5D−F** and **7**, in both cases, hydroxyl groups of carnosol interacted with the wildtype lysine and mutated alanine by forming hydrogen bonds with the backbone group. Hence, the substitution did not alter the binding mode and indicated that the loop itself contributed to carnosol binding irrespective of the amino acid sidechain (**Figure 7**). In summary, our molecular docking study strongly suggests that carnosol might inhibit p300 HAT activity by blocking the entry of the acetyl-CoA binding pocket of the catalytic domain. It is noteworthy to mention that for comparison, p300 structure were also docked with known HAT inhibitors C646 and CPTH2. The HAT residues these molecules interacted with as well as the GlideScore and MM-GBSA binding energy values obtained are reported in **Table 2**.

### Carnosol Inhibits p300 Acetyltransferase Activity by Directly Competing With Acetyl CoA *In Vitro*

**Figure 8:**
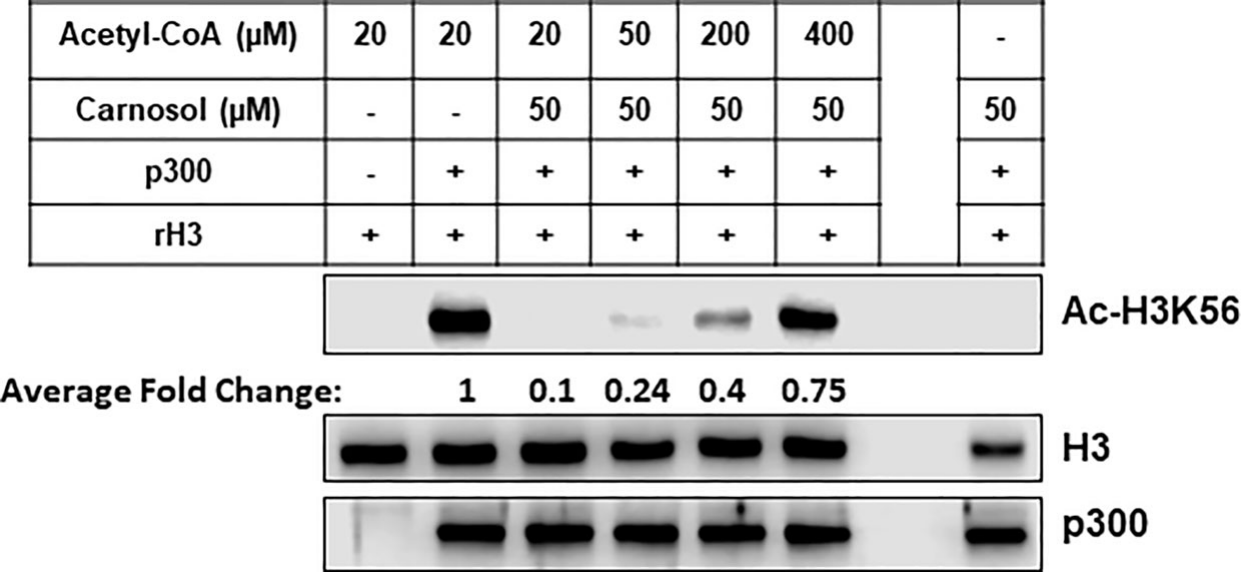
Carnosol competes with acetyl-CoA to bind to the p300 catalytic domain. Recombinant p300 catalytic domain (aa 1,284–1,673) was incubated with recombinant histone H3 and increasing concentrations of acetyl-CoA in presence of 50 µM carnosol. Histone acetylation was monitored by Western blotting scoring of acetylated H3K56. Data shown for all HAT assays are representative of three independent experiments. The intensities of the bands were quantified using the ImageJ software (National Institute of Health, USA).

To confirm the *in silico* findings, we carried out an *in vitro* HAT assay with carnosol, recombinant histone H3 and increasing concentrations of acetyl-CoA. As shown in **Figure 8**, increasing concentration of acetyl-CoA up to 400 μM led to recovery of acetylation of H3K56 by p300. These data, together with the molecular docking data, demonstrates that carnosol inhibits p300 acetyltransferase activity by directly competing with acetyl-CoA binding within the acetyl-CoA binding pocket of the catalytic domain of p300.

## Discussion

Here, we report that carnosol, a natural polyphenol and major constituent of sage, rosemary, and oregano induced histone hypoacetylation in MDA-MB-1231 and Hs578T breast cancer cells. In addition, carnosol inhibited p300 acetyltransferase activity *in vitro* and promoted its ROS-dependent proteasome degradation along with PCAF. Finally, we report that carnosol inhibits the acetyltransferase activity of p300 by obstructing the entrance of acetyl-CoA binding pocket of the catalytic domain.

p300, a transcriptional coactivator and histone acetyltransferase, consists of several conserved domains including acetyl lysine binding bromodomain, zinc binding domain and a centrally located HAT domain. These domains directly interact with several cellular proteins and transcriptional factors (50). The p300 HAT domain is organized as a central *β*-sheet comprising of seven β-strands surrounded by nine *α*-helices and several loops. This domain can further be split into two subdomains, a smaller C subdomain that comprises three *α*-helices and one *β*-strand that spans the entire structure by capping the opposite ends of the larger N subdomain (51). The acetyl-CoA binding site of p300 resides in a deep hydrophobic cavity that extends into the interior of the protein. The back of the hydrophobic cavity is lined by residues Ile1395, Tyr1397, Leu1398, and Ile1435. The larger number of interactions and better binding energy of acetyl-CoA could be attributed to its size. Among HATs, the presence of an unusually long substrate-binding loop, L1, located between *α*4 and *β*5 is a feature unique to p300 and appears to encapsulate acetyl-CoA. In all the acetyl-CoA structures of p300, Arg1410 formed hydrogen bonds with the phosphates of acetyl-CoA. The significance of this interaction has been established by mutagenesis and inhibition studies using 3′-dephospho-Lys-CoA (52). Carnosol occupied the same region where the pantetheine arm of acetyl-CoA bound. Both the lactone moiety and pantetheine arm made extensive interactions with the long loop, L1. This extensive interactions in turn permits the L1 loop to make intramolecular interactions with other regions of the HAT domain of p300 (50). Carnosol is positioned in the substrate-binding tunnel of p300 and would clash with the incoming substrate. A lysine in the substrate-binding loop was found to contribute the most interaction energy in the p300-carnosol complex. Altering this to an alanine did not cause any significant change in the binding pose and energy indicating the significance of the involvement of whole loop in the binding of carnosol in p300. Finally, carnosol binds to p300 with a better binding energy than the p300-specific inhibitor C646 and the HAT inhibitor CPTH2.

It is well documented that p300 functions as a coactivator of multiple transcription factors to regulate the expression of large number of genes controlling many biological processes (53, 54). Also, the HAT activity of p300, which is weak intrinsically, is stimulated by autoacetylation of some residues located in the HAT domain (55). Here, we showed that in addition to targeting p300 to degradation, carnosol inhibits the acetyltransferase activity required for autoacetylation and acetylation of target proteins. Our *in silico* molecular docking data along with competition assay (**Figure 8**) suggest that carnosol inhibits p300 activity by blocking the entry of acetyl-CoA into the active site and hence its autoacetylation. Several observations suggest that the HAT activity of p300 is essential for its stability. Work by Jain and collaborators showed that autoacetylation is also essential for p300 stabilization during oxidative stress (56), thus suggesting that autoacetylation of p300 not only stimulates p300 transactivation function but also contributes to its stabilization. Indeed, these authors showed that treatment of rat cardiac myocytes with the ROS inducer, doxorubicin, led to a significant stabilization of p300. Remarkably, p300 stabilization, which renders this protein resistant to proteasome-degradation, was entirely due to an increase in the acetylation of p300 that was dependent on its HAT activity. The half-life of p300 decreased in the presence of the inhibitor of p300 anacardic acid (56). Also, curcumin, a selective inhibitor of p300 HAT activity and inducer of oxidative stress, was reported to promote the proteasomal-dependent degradation of p300 (35). Based on these observations, it become tempting to hypothesize that the lack of p300 autoacetylation, due to the inhibition of HAT activity, combined with an oxidative stress could account, although possibly not solely, for the targeting of p300 protein to proteasomal degradation. We had previously reported that carnosol induced a dramatic generation of ROS in Hs578T (38) and MDA-MB-231 (39) cells. Here we reported that carnosol not only inhibited p300 HAT activity, but also promoted its proteasome degradation *via* ROS generation. We showed that blockade of proteasomal activity or ROS generation restored p300 protein that lacks HAT activity. Indeed, rescue of p300 was not associated with restoration of acetylation of its preferred substrate, H3K56. Altogether, our data support the hypothesis that one possible mechanism through which carnosol targets p300 to degradation involves the inhibition of p300 autoacetylation combined with an induction of oxidative stress in breast cancer cells.

An increasing body of evidence indicates that p300 is a positive regulator of cancer progression and is related to tumorigenesis of various human cancers including the invasive MDA-MB-231 breast cancer (16, 57–59). Knockdown of p300 has been shown to reduce proliferation and cause an increase of caspase-dependent apoptosis of both intrinsic and extrinsic apoptotic pathway in prostate cancer cells (9). Also, cellular invasion was decreased upon p300 depletion and was associated with reduced MMP-9 transcription (9). Kim and collaborators showed that MMP-9 activation requires the requires p300 and that LYR71, a derivative of trimeric resveratrol, reduced MMP-9 transcripts by blocking p300 recruitment and deacetylating histone H3 and H4 on the MMP-9 promoter (60). Interestingly, we recently showed that carnosol inhibited the invasion potential MDA-MB-231 by downregulating MMP-9 and targeting STAT3 to proteasome degradation (38). Herein we showed that carnosol dramatically reduced the level of p300 in MDA-MB-231 breast cancer cells. It is possible that p300 degradation contributes to the downregulation of MMP9 and thus, inhibition of invasion of MDA-MB-231 cells.

The role of PCAF in tumorigenesis is rather controversial and seems to depend on the type of cancer. PCAF was reported to function as tumor suppressor in the case of Hepatocellular carcinoma (23, 61), gastric cancer (62), lung adenocarcinoma (63) and colorectal cancer (64) while, in other cancers such as medulloblastoma and glioblastoma it contributes to cytoprotective events that help cancer cells to survive, evade executing cell death programs and invade other tissues (65). There are only few studies carried out on the role of PCAF in breast cancer. PCAF-mediated acetylation at H3K9 at the multi drug resistance (MDR) genes was shown to contribute to the multidrug resistance of breast cancer and, knock down of PCAF significantly sensitize these cancer cells to anticancer therapeutics (66). Park et al. (67) showed that pharmacological inhibition of PCAF by N-Acylanthranilic Acids (67). Recently, Song et al. showed that Acetylation of ARF6 GTPase-activating protein ACAP4 at Lys311 by PCAF is essential for the CCL18-elicited cell migration and invasion in MDA-MB-231and MDA-MB-468 cancer breast cancer cells. Inhibition of PCAF activity by C146 (PCA inhibitor) abolished the acetylation of ACAP4 and consequently impaired the migratory and invasive ability of MDA-MB-231 cells. Similar inhibition was observed in PCAF-depleted MDA-MB-231 cells (68). The inhibition of the activity of both p300 and PCAF acetyltransferases was also shown to exert an anti-breast cancer effect. Indeed, anacardic acid (AA), an inhibitor of both PCAF and p300 activity (31), induced cell cycle arrest, apoptotic cell death in MDA-MB-231 cells (69). In addition, it was also reported that AA efficiently and significantly inhibited cell migration and invasion and downregulated the expression of MMP-9 in MDA-MB-231 cells (69, 70). Here we showed for the first time that carnosol targets PCAF to ROS-dependent proteasome degradation in breast cancer cells which contribute, at least partly to its anticancer activity. The molecular mechanism by which PCAF is targeted to degradation is yet to be uncovered. Our data, in agreement with the previous findings, strongly suggest that PCAF contributes to breast cancer survival and/or metastasis and hence represents a rational target for breast cancer therapy.

In summary, our data identifies carnosol as novel natural p300-specific inhibitor with a promising therapeutic potential against aggressive breast cancer. We hypothesize that carnosol inhibits HAT activity of p300 blocking its autoacetylation, essential for its function and stability. ROS generation contribute to the targeting of p300 and PCAF to proteasome degradation. The relevance of PCAF degradation in cancer cells remain to be explored. Finally, it is worth mentioning that carnosol is a pharmacologically safe compound with no side effects observed in mice treated for five days with doses as high as 200 mg/kg (71).

## Data Availability Statement

The original contributions presented in the study are included in the article/**Supplementary Material**. Further inquiries can be directed to the corresponding author.

## Author Contributions

HA, HH, AA, and YD performed the Western blot experiments. RI and AR performed the HAT assays. BB and RV performed the *in silico* docking analysis. KH and HS performed the migration assay and measurement. MA performed the RT-PCR and qRT-PCR experiment. AE and RI helped analyzing the data. RI designed the project and wrote the manuscript. All authors contributed to the article and approved the submitted version.

## Funding

This work was supported by the Zayed Center for Health Sciences (ZCHS) research grant (Grant # 31R086) and by Al Jalila Foundation Research Grant (Grant # 21S102-AJF2018007) to RI.

## Conflict of Interest

The authors declare that the research was conducted in the absence of any commercial or financial relationships that could be construed as a potential conflict of interest.
