# Supplementary figures and images for: Carnosol Is a Novel Inhibitor of p300 Acetyltransferase in Breast Cancer
Source: Front Oncol. 2021 May 13;11:664403. doi: 10.3389/fonc.2021.664403 (PMC8155611; doi:10.3389/fonc.2021.664403)

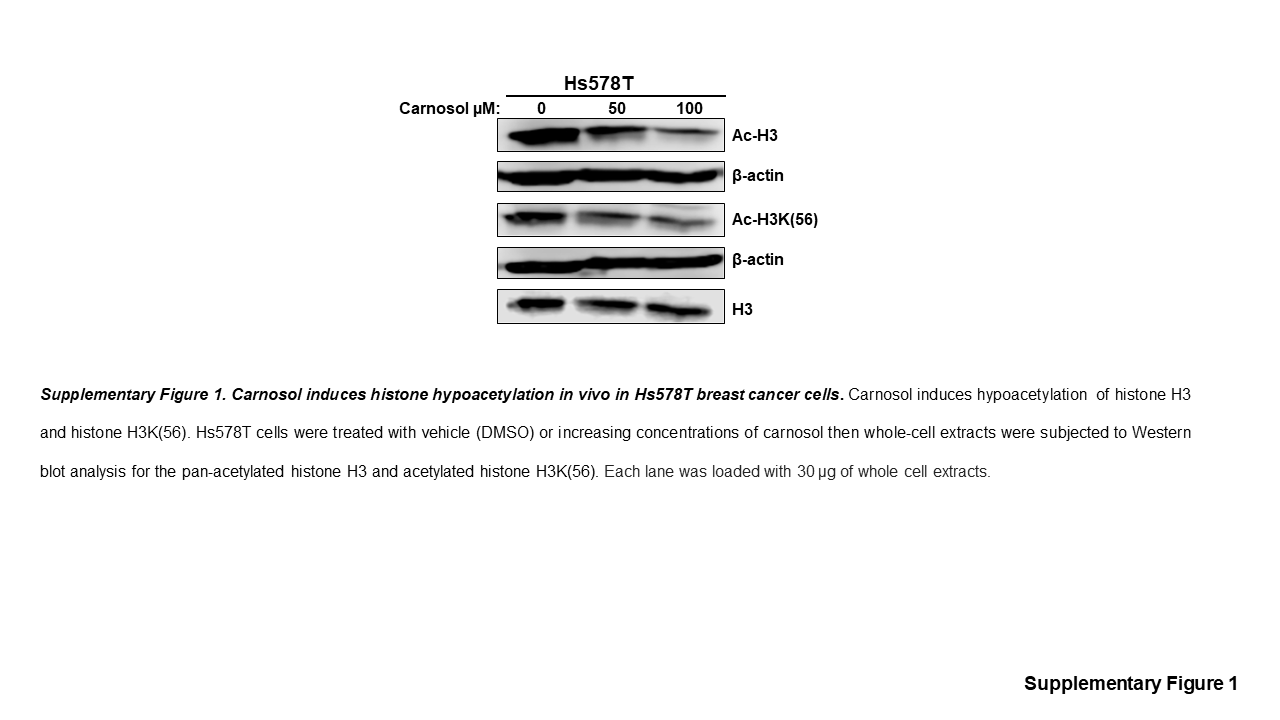

Supplement: Supplementary file 1 [file Image_1.tif]

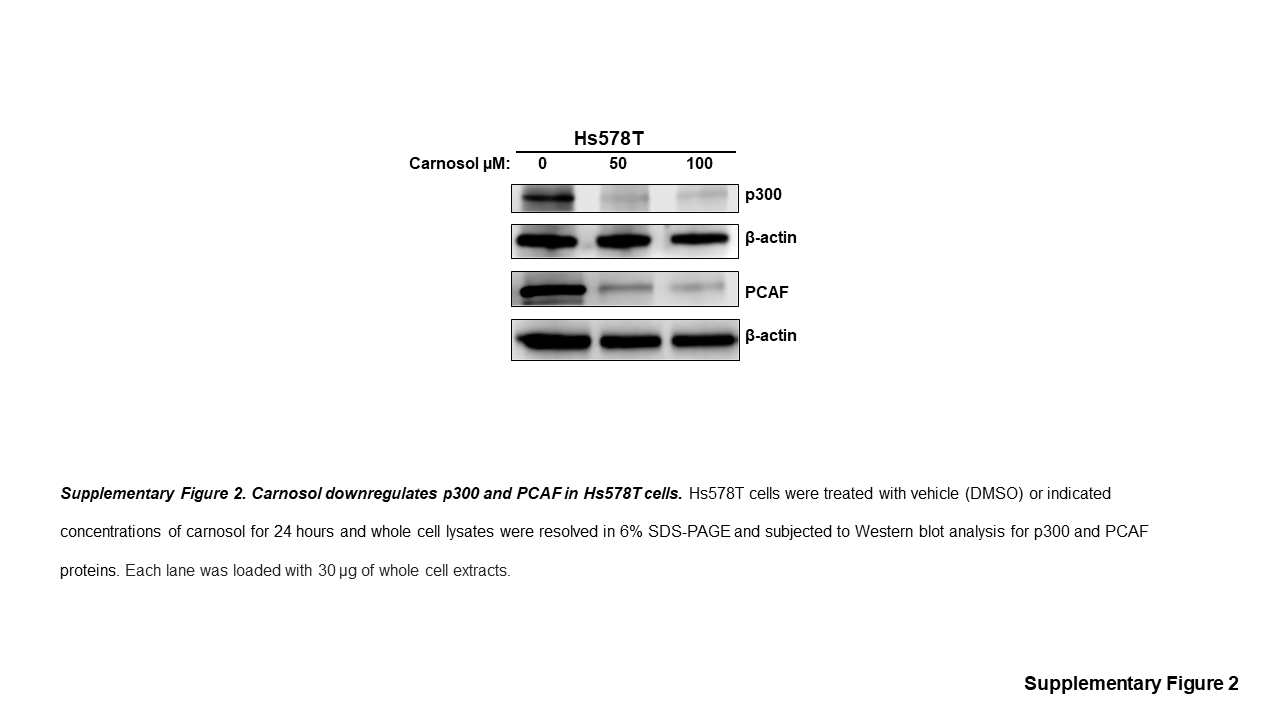

Supplement: Supplementary file 2 [file Image_2.tif]

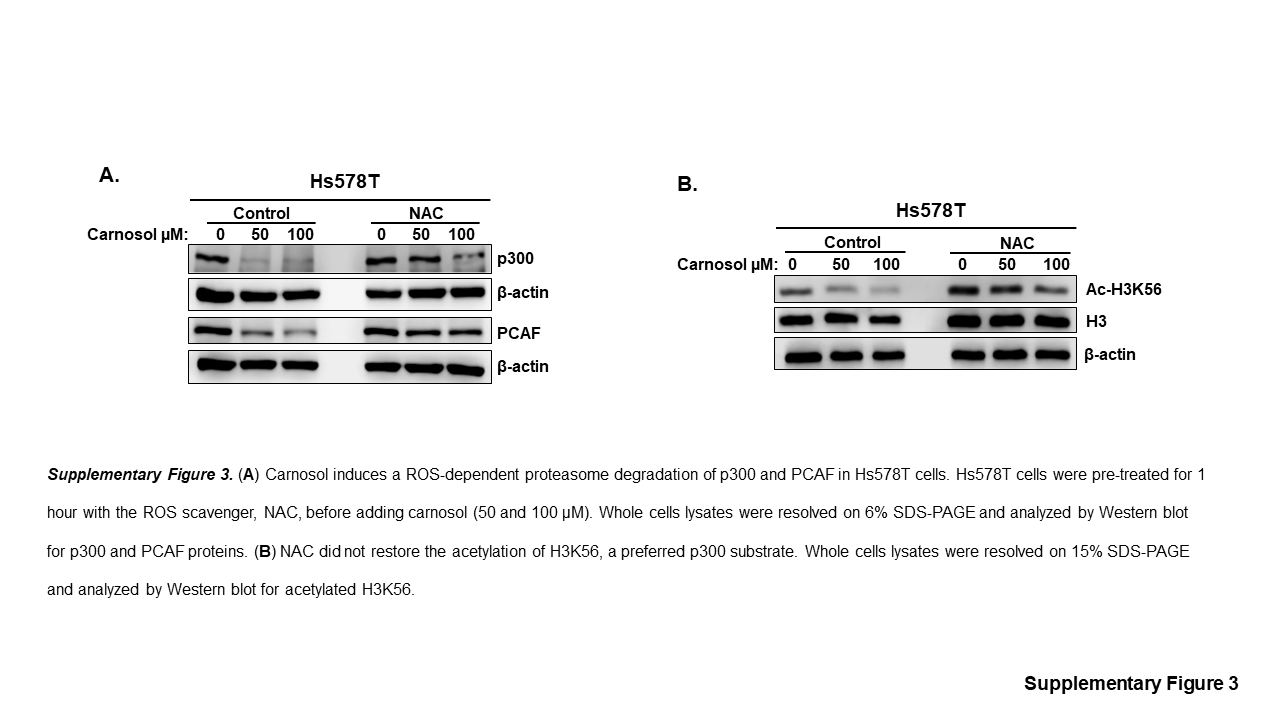

Supplement: Supplementary file 3 [file Image_3.tif]

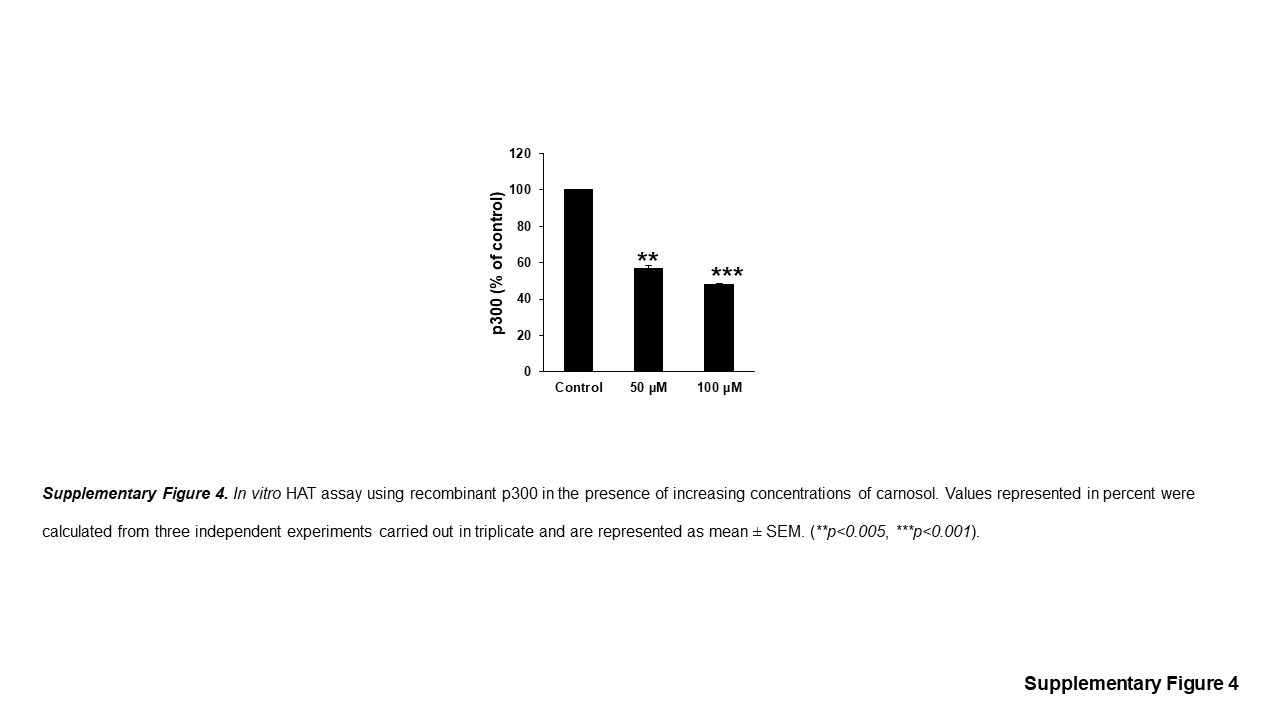

Supplement: Supplementary file 4 [file Image_4.tif]
